# Enzymes and Mechanisms Employed by Tailed Bacteriophages to Breach the Bacterial Cell Barriers

**DOI:** 10.3390/v10080396

**Published:** 2018-07-27

**Authors:** Sofia Fernandes, Carlos São-José

**Affiliations:** Research Institute for Medicines (iMed.ULisboa), Faculty of Pharmacy, Universidade de Lisboa, Av. Prof. Gama Pinto, 1649-003 Lisboa, Portugal; as.fernandes@ibet.pt

**Keywords:** bacterial cell envelope, proton motive force, cell wall, peptidoglycan, depolymerase, endolysin, lysin, holin, spanin, LysB

## Abstract

Monoderm bacteria possess a cell envelope made of a cytoplasmic membrane and a cell wall, whereas diderm bacteria have and extra lipid layer, the outer membrane, covering the cell wall. Both cell types can also produce extracellular protective coats composed of polymeric substances like, for example, polysaccharidic capsules. Many of these structures form a tight physical barrier impenetrable by phage virus particles. Tailed phages evolved strategies/functions to overcome the different layers of the bacterial cell envelope, first to deliver the genetic material to the host cell cytoplasm for virus multiplication, and then to release the virion offspring at the end of the reproductive cycle. There is however a major difference between these two crucial steps of the phage infection cycle: virus entry cannot compromise cell viability, whereas effective virion progeny release requires host cell lysis. Here we present an overview of the viral structures, key protein players and mechanisms underlying phage DNA entry to bacteria, and then escape of the newly-formed virus particles from infected hosts. Understanding the biological context and mode of action of the phage-derived enzymes that compromise the bacterial cell envelope may provide valuable information for their application as antimicrobials.

## 1. Introduction

Viruses are obligatory intracellular parasites, meaning that they need to infect suitable cells to multiply and proliferate. When they exist outside host cells, viruses are normally inert entities called virus particles or virions. These supramolecular structures are optimized to protect the viral genetic material from environment insults and to ensure its efficient transmission to host cells. A typical infection initiates with the virion recognizing and binding to at least one specific receptor of the host cell surface. This is followed by the viral genome routing to the site of replication within host cells, the expression of this genome to form tens or hundreds of new virus particles, and finally virion progeny escape to the extracellular space. Therefore, during an infection cycle, viruses need to overcome the host cell envelope at least twice, first to get inside cells and then to escape from them after virus multiplication [1,2,3]. The cell barriers to virus entry and exit are basically determined by the composition and structure of the host cell envelope, varying from “simple” lipid membranes in animal cells, to complex multilayered envelopes that include membranes and polymeric cell walls in plants, fungi and bacteria. The host cell barriers shape to great extent the virion properties required for viral genome delivery to the cell, and the pathways and enzymatic machineries necessary for efficient liberation of the virus descendants from infected cells [1,2,3].

Bacteriophages, or phages, are the viruses that infect bacteria. They are frequently described as the most abundant and diverse biological entity on Earth, being virtually present in every place where bacteria thrive, and playing a major role on their dynamics and evolution [4,5]. The virus particle of around 96% of the studied phages is made of an icosahedral capsid enclosing a linear double-stranded DNA (dsDNA) genome and a tail structure attached to one vertex of the nucleocapsid (tailed phages [6]). This megadalton virion structure cannot penetrate the bacterial cell envelope (BCE), which in its minimal configuration is composed of a cytoplasmic membrane and a cell wall made of peptidoglycan [7]. Therefore, to initiate infection phages need to surgically perforate the different structures of the BCE to deliver just the genetic material, and eventually a few proteins, into host cells. As it will be explained in Section 3, the tail apparatus has an essential role in this process. On the other hand, massive host cell lysis is usually required to allow efficient escape of the newly-formed virus particles at the end of infection. In fact, tailed phages have developed functions and strategies to promote abrupt and concerted disruption of the different BCE layers at the appropriate time for virion progeny release (Section 4).

One interesting feature that is common to the virus entry and lysis steps is the use by phages of enzymes that compromise the different host cell barriers. These enzymes have been intensively studied due to their potential applications in bacterial detection and elimination in different contexts, including medicine, food industry and agriculture [8]. Those degrading the cell wall peptidoglycan (virion-associated lysins and endolysins) have been receiving particular attention, with a great number of in vitro and in vivo studies supporting their possible use as alternatives to antibiotics [9,10,11,12,13]. Knowledge on the phage enzymes that attack the BCE can be used to devise new antibacterial agents. Therefore, we present here the major features of this group of enzymes and their mode of action in the context of phage infection, with particular emphasis on those that can be explored as enzybiotics to treat infections caused by multidrug resistant bacteria.

## 2. The Barriers of the Bacterial Cell Envelope

**Figure 1:**
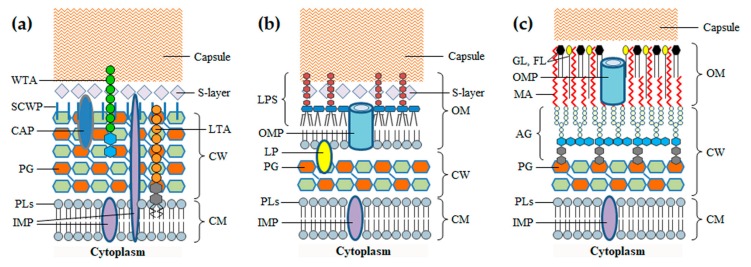
Schematic representation of the structure and composition of the bacterial cell envelope in Gram-positive bacteria (**a**), Gram-negative bacteria (**b**) and mycobacteria (**c**). CM, cytoplasmic membrane; CW, cell wall; OM, outer membrane; IMP, inner membrane proteins; PLs, phospholipids; PG, peptidoglycan; AG, arabinogalactan; LP, lipoprotein; CAP, covalently attached protein; LTA, lipoteichoic acids; SCWP, secondary cell wall polymers; WTA, wall teichoic acids; OMP, outer membrane protein; LPS, lipopolysaccharide; MA, mycolic acids; GL, glycolipids; FL, free lipids. The S-layer and capsule are extracellular structures. Branched lipoaraninomannan is not represented in the mycobacterial cell envelope (probably anchored to both the CM and OM).

Before addressing the phage-derived enzymes that attack the BCE, it is important to highlight the main features of this cellular structure, namely regarding its composition and key biological functions. The BCE is a complex multilayered structure that needs to preserve a minimum level of integrity to guarantee cell survival. It secures cell homeostasis by protecting from environment insults, allowing at the same time the influx of nutrients and the efflux of waste products to sustain growth [7,14]. The simplest BCE is composed of a cytoplasmic membrane (CM) and a rather thick cell wall (CW). This type of BCE is characteristic of Gram-positive bacteria, which are also considered monoderm for having just one membrane. Gram-negative bacteria and mycobacteria are diderm because they have an additional membrane, the outer membrane (OM), surrounding the CW. In addition to the BCE, bacteria may produce proteinaceous S-layers and polysaccharidic capsules, which form the outermost coats encasing the cell (Figure 1). Protein apparatus like flagella, pili and specialized transport systems may be attached to the BCE, some of which spanning and protruding from the cell surface.

The CM is common to all bacteria, functioning as a semipermeable barrier that separates and protects the cytoplasmic content from the extracellular environment. It is a symmetric bilayer of phospholipids with embedded glycolipids, lipoproteins, proteins and protein complexes, which perform fundamental roles in cell survival such as nutrient uptake, sensing and response to environmental stimuli, and biogenesis of the BCE itself [14]. Many Gram-positive bacteria have lipoteichoic acids (LTA) anchored to the CM via a glycolipid anchor, with a phosphate-rich polymeric moiety extending through the CW [15]. Central to the subject of this review is the CM function in energy production. Electron carriers and transporters in the CM are responsible for the formation of ion gradients (notably of protons) across the membrane. The differences in the charges and concentration of ions on opposite sides of the CM generate, respectively, the electric (ΔΨ) and the chemical potential (proton gradient, ΔpH), the two components of the so-called proton motive force (PMF). The PMF is involved for example in the generation of adenosine triphosphate (ATP) through oxidative phosphorylation, control of bacterial autolysis, glucose transport and cell motility, and its irreversible collapse leads to bacterial cell death [16,17].

The CW is responsible for maintaining cell shape and integrity by counteracting the high cytoplasmic osmotic pressure. This organelle is nonetheless dynamic enough to allow cellular extension and division [18]. The major structural component of the CW is peptidoglycan (PG), also known as murein, which forms a polymeric network surrounding the CM. The PG layers may be decorated with numerous proteins that associate with the murein either via electrostatic interactions or through covalent binding. In Gram-positive bacteria the PG is also covalently modified with carbohydrate polymers, frequently with anionic wall teichoic acids (WTA) [19]. The thick CW core of mycobacteria is composed of PG covalently attached to the arabinogalactan network, which in turn forms covalent bonds with mycolic acids of the OM (see below) [20].

**Figure 2:**
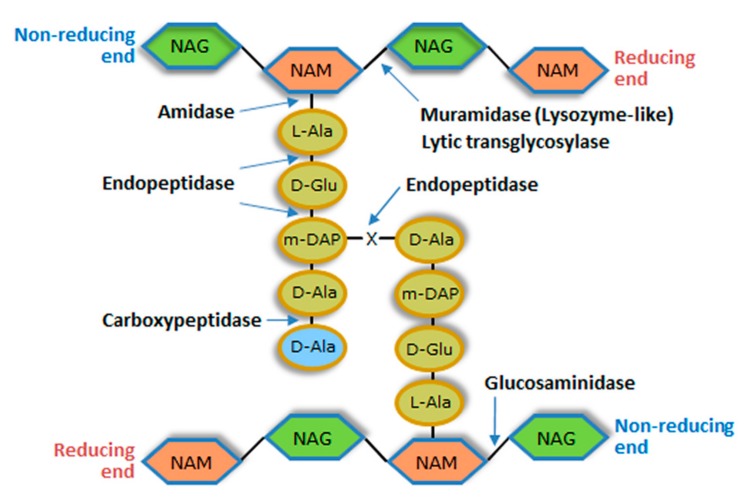
Basic structure of the bacterial cell wall peptidoglycan (PG). The possible enzymatic activities of PG-degrading enzymes and the bonds they cleave are indicated. m-DAP is found in the peptide chains of the PG of most Gram-negative bacteria, *Bacillus* spp. and *Listeria* spp., which present also direct m-DAP-d-Ala bonding between adjacent stem peptides. In most Gram-positive bacteria m-DAP is replaced l-Lys. Cross-linking between this residue and d-Ala of a neighbor peptide chain usually occurs by an interpeptide bridge of variable amino acidic composition (X). The d-Ala residue in light blue may be lost after PG maturation. Carboxypeptidases are rarely produced by bacteriophages. NAG, *N*-acetylglucosamine; NAM, *N*-acetylmuramic acid.

Each PG layer corresponds to linear glycan chains having as repeating unit a disaccharide made of *N*-acetylglucosamine (NAG) and *N*-acetylmuramic acid (NAM), linked by glycosidic bonds β (1 → 4). Adjacent glycan strands are cross-linked by short peptides that are attached to NAM via amide bonds [21]. Typically, cross-linking involves the amino acid residues at positions 3 (often l-Lys or meso-diaminopimelic acid (m-DAP)) and 4 (d-Ala) of complementary peptide chains. These peptide stems may be linked by a direct interpeptide bond (most Gram-negative bacteria and few Gram-positive species) or via an interpeptide bridge (most Gram-positive bacteria). The different types of bacterial PG are mainly defined by variations within the peptide moiety, particularly in the amino acidic composition of the interpeptide bridges. In Gram-positive bacteria the multiple layers of PG can make the CW 10-fold thicker than that of Gram-negative bacteria [21,22] (Figure 2).

The CW murein is targeted by a diverse group of degrading enzymes of endogenous or exogenous origin. Those produced by bacteria are involved in several key functions that include defence against competing bacteria, cleavage of the cell divisional septum, and PG maturation, turnover and recycling [23]. Deregulation of endogenous PG-degrading activity can result in bacterial autolysis, and the enzymes responsible for this phenomenon are called autolysins [17]. Besides bacteriophages, almost all living organisms synthesize enzymes that attack the bacterial PG. In animals for example, lysozyme is a key element of innate immunity [24]. Irrespective of their origin, PG-degrading enzymes can be classified into three major groups according to the bond they cleave in the murein network: glycosidases, amidases and peptidases. Glycosidases cleave one of the two glycosidic bonds in the glycan chain and can be subdivided into *N*-acetyl-*β*-d-glucosaminidases (glucosaminidases), *N*-acetyl-*β*-d-muramidases (muramidases or lysozymes) and lytic transglycosylases (Figure 2). PG amidases hydrolyze the amide bond between the first amino acid residue of the peptide stem (generally l-Ala) and NAM (*N*-acetylmuramoyl-l-alanine amidases), while peptidases cleave within or between the peptide strands (Figure 2). Peptidases are subdivided into carboxypeptidases, which remove C-terminal amino acid residues, and endopeptidases that cleave internal bonds of the peptide. All these enzymes seem to break the PG though a hydrolysis mechanism, except lytic transglycosylases [23].

The OM works as an extra lipid barrier that surrounds the CW and, together with the CM, it establishes the cell compartment called periplasm. In Gram-negative bacteria the OM corresponds to an asymmetric bilayer, in which the inner leaflet is composed of phospholipids and the outer leaflet of glycolipids, notably, lipopolysaccharides (LPS). The LPS molecule is composed of three portions: the lipid moiety (lipid A), a nonrepeating “core” oligosaccharide and the highly variable *O*-antigen polysaccharide [25]. The LPS *O*-antigen is one of the major surface antigens contributing to serotype differentiation in Gram-negative bacteria and it frequently works as receptor for phages infecting this type of host cells [26]. There are two main types of proteins inserted in the OM, the integral outer membrane proteins (OMPs, many working as *β*-barrel transporters) and lipoproteins, as for example the Braun’s lipoprotein that covalently anchors the OM inner leaflet to the PG layer [25]. The structure and composition of the OM in mycobacteria is more complex and less understood than that of Gram-negative bacteria. The mycolic acids of the inner side of the mycobacterial OM are covalently attached to the arabinogalactan network of the CW. Several glycolipids, phospholipids and free lipids, some of which are species-specific, distribute through the OM bilayer conferring an overall symmetrical appearance. The OM is also spanned by pore-forming OMPs, but apparently in low numbers when compared to Gram-negative bacteria [20,27,28]. Outside of the mycobacterial OM is a layer of proteins, polysaccharides and some lipids, which collectively form a coat known as the capsule ([29] and references therein).

## 3. Crossing the Bacterial Cell Envelope to Get Inside: A Tale of Surgical Tails

The molecular details underlying BCE penetration by phages are still poorly characterized when compared to our current understanding of the mechanisms mediating phage release from infected cells (Section 4). It is well established nevertheless that the phage tail plays a critical role in the entry process by governing two major events: (i) the search and binding (adsorption) to receptors of the BCE surface, and (ii) the controlled perforation of the BCE and channeling of the viral DNA into the host cell cytoplasm [30,31]. Adsorption involves the specific interaction between tail receptor-binding proteins (RBPs) and one or more BCE receptors. RBPs are major determinants of phages’ host-range and can localize in different substructures of the tail, namely fibers, spikes or baseplates [32,33,34,35]. Host cell surface receptors are usually components of the outermost layers of the BCE, such as proteins, sugars and different structural (LPS, WTA, LTA, PG) and extracellular (capsules, S-layer) polymers, but can also be flagella and pili [26,36]. Interestingly, upon binding some RBPs enzymatically cleave or modify the polymeric moieties of the corresponding receptors, like for example the LPS and capsules of Gram-negative bacteria [26,37], and the WTA of Gram-positive hosts [38,39,40] (see Section 3.1).

A paramount feature of the initial steps of phage infection is that upon appropriate tail/receptor interactions the bound virion suffers major structural rearrangements. This happens because free virions store energy in a metastable state and interaction with the bacterial receptors under suitable conditions triggers the transition to a structural configuration of lower energy [41]. This transition is crucial to open the head-to-tail connector for DNA exit and to allow tails to form a conduit across the BCE for channeling of the viral genome into host cells [31,32,33,34]. As detailed in Section 3.2, formation of the transenvelope channel frequently involves protrusion and insertion in the BCE of tail substructures, some of which are endowed with PG-degrading activity to facilitate crossing of the CW barrier. The tip of the penetrating tail tube may directly fuse with the CM or eventually interact with host cell membrane channels ([42] and references therein), which is finally followed by phage DNA translocation (for a review on the forces and mechanisms driving DNA movement from the phage head to the bacterial cytoplasm see [43]).

### 3.1. Phage Depolymerases

**Figure 3:**
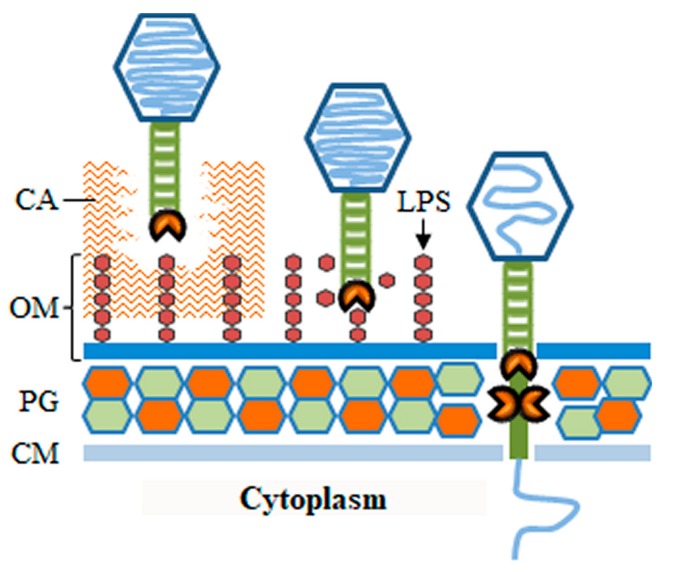
Possible actions of phage depolymerases during recognition and penetration of the bacterial cell envelope (Gram-negative bacteria used as example). Depolymerase activity is generically depicted as a pacman symbol. CM, cytoplasmic membrane; PG, cell wall peptidoglycan; OM, outer membrane; LPS, lipopolysaccharide; CA, capsule.

As referred to above, the phage virus particle, particularly the tail apparatus, may be equipped with enzymes that degrade polymeric components of the bacterial surface. These enzymatic activities may be important for the phage to go through protective substances that block the access to cell receptors, to mediate the actual process of receptor binding, or to overcome barriers of the BCE such as the CW (Figure 3). Most of these enzymes belong to a broad group of polysaccharide-degrading proteins, also known as polysaccharide depolymerases. Depolymerases may degrade extracellular polysaccharides (EPS), such as those composing capsules or biofilm matrices, or cleave structural polysaccharides like the LPS or the PG glycan stands. Since these enzymes target and compromise structures important for cell survival and virulence they may found applications in the control of bacterial pathogens [44,45,46,47]. The only virion-associated depolymerases capable of provoking host cell lysis are those targeting the PG and for this reason they are also included in the group of virion-associated lysins [47]. These and other PG-degrading enzymes (e.g., peptidases) carried in the virus particle are separately addressed in Section 3.2.

As already mentioned, the most external structure in some bacteria may correspond to a capsule that completely encases the cell. Most capsules are composed of polysaccharides and in pathogenic bacteria they can be major virulence factors that impair host immune recognition/inactivation and phagocytic killing [48,49]. Although capsules may protect bacteria from phage infection by masking host cell receptors [50], several phages have evolved to use these polysaccharidic coats as receptors to initiate infection, with several examples particularly known in Gram-negative systems. These phages typically carry several copies of one or more types of tail fibers or tailspikes, each targeting and degrading a certain capsular type [51,52,53,54]. Thanks to their processive depolymerase activity (sequential cleavage of polymer bonds without virus dissociation [51]), these tail structures allow virions to drill through the capsule layer until eventually reaching secondary receptors in the BCE that trigger subsequent steps of the viral DNA entry process. In addition, phages may encode EPS depolymerases to facilitate penetration of biofilm matrices and infection of resident bacteria [44,55,56]. When plated in soft agar medium, phages with depolymerase activity often produce transparency halos in the bacterial lawn that surrounds phage plaques. These halos result from diffusion of the depolymerase activity (virions and/or free enzymes [55]).

The *O*-polysaccharide chain of LPS is another type of receptor that is frequently hydrolyzed by virion depolymerases of phages infecting Gram-negative bacteria. The best characterized LPS-hydrolyzing RBPs are the tailspikes of podoviruses (short-tailed phages) [57,58,59]. These tailspikes were initially thought to allow phages to move through the LPS layer until reaching a secondary or irreversible receptor in the OM (e.g., OMPs), which would trigger opening of the virus particle for DNA ejection [36]. Several studies however have shown that different *O*-antigen specific phages are unable to infect host cell “rough” variants lacking the *O*-antigen portion of the LPS. In fact, tailspike binding and cleavage activities seem to be necessary not only for positioning the phage particle on the OM, but also to prime its opening ([37] and references therein). In good correlation with this, it was shown that highly purified LPS can induce phage DNA ejection in vitro when incubated with virions having this type of tailspikes [60,61,62].

It is outside the scope of this review to present a comprehensive view of the highly diverse phage-encoded depolymerases. A recent in silico survey performed by Pires et al. [46] found 160 putative depolymerases in 143 fully sequenced phages infecting 24 genera of bacteria. According to the mechanism of polysaccharide cleavage, the enzymes can be classified as hydrolases or lyases. The first group includes for example sialidases that degrade capsular polysialic acid, well characterized in phages infecting *Escherichia coli* of the K1 capsular type [63], and rhamnosidases that hydrolase the LPS *O*-antigen [57,58]. Depolymerases with phosphoesterase activity towards WTA are also hydrolases [39,40]. Lyases include the hyaluronidases that cleave hyaluronate-based capsules and EPS [64], the pectate/pectin lyases responsible for degradation of biofilm EPS [65], and the alginate lyases encoded by some *Azobacter* and *Pseudomonas* phages that degrade the alginic acid EPS of host cells [66,67]. Some virion-associated depolymerases cleave polypeptide or lipid substances instead of polysaccharides, which is probably a response to the particular nature of some bacterial extracellular structures [46]. Recent and excellent reviews provide a compilation of the phages producing depolymerases and their location in the virion structure, as well as a detailed view of their diversity and enzymatic properties [44,45,46,47].

### 3.2. Virion-Associated Lysins

As highlighted in the previous section, some phages may employ tail depolymerases to clear the path for virions to reach the cellular surface. Here, irreversible binding to host receptors induces key changes in the virion structure for opening of the nucleocapsid and for tail insertion in the BCE [31]. As knowledge progresses in this area it is becoming clear that, irrespective of tail morphology, many phages need to eject proteins or protrude tail substructures to extend their tails and span the BCE [42]. While crossing the membrane layers of the BCE may be achieved by mechanical puncturing and/or protein fusion with the lipid constituents, traversing the rigid CW may be more challenging. In fact, some of the virion proteins that insert in the BCE are known to have PG-degrading activity, probably to facilitate tail CW crossing (Figure 3). These proteins will be referred here as virion-associated lysins (VALs), but they are also known in the literature as virion-associated peptidoglycan hydrolases (VAPGH), tail-associated muralytic enzymes (TAME), tail-associated lysins (TAL), exolysins, or structural lysins [47,68]. To maintain host cell integrity during virus entry, the CW murein should be degraded by VALs only locally. In fact, when infecting bacteria at high multiplicities some phages can cause the phenomenon of “lysis from without”, in which host cells lyse immediately as result of VAL-mediated PG degradation at multiple sites [69].

VALs most often correspond to individual components or to domains of tail proteins, like the tape measure protein (TMP), central fibers, tail tip knobs, and tail tip puncturing devices, but they can also be capsid inner proteins that are ejected upon virus opening [47,68]. A remarkable example of virion structure dynamics and VAL action is provided by *E. coli* phage T4 and its contractile tail (myovirus). Irreversible binding to host receptors induces tail sheath contraction, causing the inner tail tube with a puncturing device at its tip to penetrate the BCE. One of the proteins composing the piercing apparatus is gp5, which has muralytic activity [34,70]. For the entry process some phages with long, non-contractile tails (siphoviruses) are known to eject and insert in the BCE the internal tail tube formed by the TMP [33,71]. In some siphoviruses (e.g., the *E. coli* phage T5 and the mycobacteriophage TM4) the TMP was shown to carry PG-cleaving domains [72,73,74]. After irreversible adsorbing to cellular receptors, the *E. coli* phage T7 (podovirus) ejects the proteins composing the capsid inner core to form an extended tail tube that spans the BCE. One of the T7 core proteins, gp16, has PG-degrading activity ([75] and references therein). Other podoviruses like φ29 simply seem to drill through the CW, helped by the PG-hydrolysis activity of one of the components of the tail tip knob [38].

Independently of the location of VALs in the virion structure and of the mechanism responsible for their positioning on the CW, it is thought that these enzymes promote a local digestion of the PG to facilitate penetration or extension of the tail tube across the CW and its subsequent fusion to the CM. Yet, for many phages it is not obvious that the virus particle is equipped with a VAL and in others (e.g., *E. coli* phage T7, mycobacteriophage TM4 and *Staphylococcus aureus* phage phi11) the presence of the lytic enzyme in the virion was shown dispensable for phage infection under laboratory conditions [72,76,77]. In fact, the available data suggests that VALs may confer an advantage when phages have to infect cells under physiological conditions promoting PG thickening and/or increased cross-linking (e.g., stationary growth, low temperature) [72,76,78].

The VAL domains responsible for PG-cleavage (catalytic domains, CDs) usually specify glycosidase or endopeptidase activity (Figure 2), with the latter found almost exclusively in phages infecting Gram-positive bacteria [47]. VALs tend to be multifunctional, large proteins and some are found as oligomers in the virus particle. As components of the virion structure, VALs may play key roles in the morphogenesis, stability and infectivity of phage virus particles [77,78]. Whereas VALs of phages infecting Gram-negative bacteria typically display a single CD, those of Gram-positive systems frequently carry two CDs, with distinct cleavage specificities, which probably reflects the thicker PG layer of the bacterial hosts (for reviews on VALs features, see [11,47]).

## 4. Crossing the Bacterial Cell Envelope to Get Outside: Knocking Down All Barriers

As seen above, phages use their tails with incorporated enzymes to surgically deliver their genetic material across the BCE without compromising cell viability. Quite oppositely, the mechanisms for virion progeny release from infected bacteria typically involve considerable destruction of the BCE, with consequent host cell burst (lysis). Tailed phages accomplish bacterial cell lysis through the concerted action of at least two phage-encoded functions, the endolysin and the holin [79,80]. Endolysins are PG-degrading enzymes that build in the cell during infection and they are instrumental to induce rapid osmotic cell lysis [3,81]. Holins are hydrophobic proteins that start to oligomerize in CM at mid or late stages of phage replication. After reaching a genetically defined threshold concentration in the CM, they are triggered to form holes that collapse the PMF, leading to immediate cell death [82,83]. In addition, in the so-called canonical lysis model that is represented by the *E. coli* phage λ, these holes are large enough to allow passage of the cytoplasm-accumulated endolysin to the CW, which is an essential requirement for occurring cell lysis [80,84]. The λ holin-endolysin lysis system was for long considered the model mechanism that would generically apply to all tailed phages (actually, to all dsDNA phages). In the year 2000, however, the first of several studies was published reporting lysis mechanisms that deviated from the λ paradigm, particularly regarding the way endolysins were targeted to the CW compartment (see Section 4.2). In addition, more recent advances have uncovered or consolidated the role of additional lysis functions that are necessary to overcome the outermost barrier of the BCE, the OM (Section 4.4). Before moving to these latest developments, it is important to present the general properties and mode of action of the two functions that seem conserved in lysis systems of dsDNA phages, endolysins and holins.

### 4.1. Properties of the Conserved Lysis Players

The phage-encoded, PG-degrading enzymes involved in cell lysis for virion progeny release accumulate in host bacteria during the course of infection. At the appropriate time for occurring lysis (see below) they attack the CW from within and are thus called endolysins. These lytic enzymes may display any of the PG-cleavage activities indicated in Figure 2, although the most commonly found CDs in endolysins specify muramidase or amidase activity [9,85]. In its simplest structure, the endolysin is a monomeric and globular polypeptide essentially corresponding to the CD. This type of endolysin is essentially exclusive of phages infecting Gram-negative bacteria. The other endolysin type has a modular configuration in which a CD-containing region is connected by a flexible linker to a cell wall binding domain (CWBD). The latter module has high affinity to a particular CW component and is responsible for tight association of the enzyme with its substrate [86]. The lytic spectrum of these endolysins is in part defined by the CWBD they carry and by the level of conservation of the targeted CW ligand among bacteria [9,85]. The endolysins of phages infecting Gram-positive bacteria are typically equipped with CWBD. It is thought that the binding module limits the diffusion of endolysins after cell lysis by remaining attached to the CW debris. This may be advantageous to minimize destruction of potential new hosts that are in the vicinity of lysed cells. Such risk should be minor with hosts having an OM shielding the CW, probably explaining the rare presence of CWBD in endolysins of phages infecting Gram-negative bacteria. Interestingly, the CD-CWBD modular configuration is also prevalent in mycobacteriophage endolysins [87], despite the presence of an OM in mycobacteria, suggesting other useful roles for the CW binding module. Probably because VAL contact to the CW is guaranteed by the tail dynamics and by the tight interaction between RBPs and host cell surface receptors, VALs seem to lack CWBD [11,47].

The endolysin modular architecture can vary significantly according to the type, number, and relative position of CDs and CWBDs. Modular endolysins composed of one or two N-terminal CDs of different cleavage specificities, which are coupled to a C-terminal CWBD module made of single or multiple copies of the same binding motif are typical of phages infecting Gram-positive hosts [9,85]. Although much less abundant, some endolysins of phages infecting Gram-negative hosts may present a modular structure, but in this case with an inverted organization of the functional domains, that is, the CWBD and the CD occupy the N- and the C- terminal regions of the enzymes, respectively ([88,89] and references therein). A few endolysins have been shown to work as hetero-oligomers, in which CD-containing subunits associate with several independently produced copies of the CWBD [90,91,92,93]. An overview of endolysins structural diversity can be found elsewhere [9,85,94,95].

Holins establish the end of phage infection by killing host cells through hole formation in the CM. They are therefore considered the clocks of phage infection [96]. In canonical lysis they also define the onset of cell lysis by providing a pathway for endolysin access to the CW [80,84]. Most of what is known about the structure and mechanism of holin hole formation derives from detailed studies with lambdoid phages, particularly with the S holin of phage λ [83]. As long as the PMF is maintained above a certain threshold, holins harmlessly accumulate and uniformly distribute in the CM during virion progeny assembly. After reaching an allele-specific critical concentration, abrupt holin nucleation and aggregation occurs with formation of “rafts” that are proposed to permeabilize the CM. PMF dissipation is then thought to suddenly cause conformational changes that allow holin rafts to convert into the final holes [83,97]. Canonical holins, such as those from phages λ, P2 and T4 form large, micron-scale holes that allow the cognate endolysins (R, K and E, respectively) to escape from the cytoplasm and rapidly degrade the PG [98,99].

To fine tune host cell lysis timing in response to infection conditions, lambdoid phages encode also a holin antagonist, which is referred to as antiholin. In some phages (e.g., λ and 21) the holin gene itself encodes both the lysis and inhibitor functions through the so-called “dual-start motif” [100]. In the case of phage λ the S holin, or S105, is a 105 aa polypeptide with three transmembrane domains (TMDs) that adopts an N-out, C-in topology. S105 results from translation initiation at the second start codon (Met3) of the S gene. The antiholin, S107, which initiates at the first start codon (Met1) of S, differs from S105 by two residues at the N-terminus, Met and Lys, which confer to the antiholin N-terminus 2 extra positive charges (one charge from the deformylated Met1 and another from the Lys2 residue [101]). These positive charges prevent insertion of the first TMD of S107 into the CM. This feature, associated to the capacity of the antiholin to dimerize with S105 is what confers to S107 its inhibitory effect. In standard laboratory conditions, a mutant phage that exclusively produces S105 efficiently lyses host cells 5 to 10 min earlier than the wild type λ. Quite remarkably, dissipation of the PMF triggers the insertion of the first antiholin TMD into the CM, adopting thus the same topology of S105 and contributing similarly to hole formation ([84,101] and references therein).

Holin and antiholin functions can be encoded by separate genes as it happens in phages T4 and P2 [102,103]. Regulation of the T4 holin T by its cognate antiholin RI provides a paradigmatic example of holin function modulation in response to an environmental signal, which in this case is produced when T4-infected cells are superinfected by other T4 or T-even phages. In the presence of this signal, the secreted RI is stabilized in the periplasm and allowed to interact with the periplasmic C-terminal domain of the single-TMD holin T, inhibiting its triggering [104,105]. This is responsible for the classic phenomenon known as LIN (lysis inhibition), in which T4 virions keep accumulating intracellularly for hours [106]. Recent evidences support that a second T4 antiholin function, RIII, also contributes to the LIN state by interacting with the cytoplasmic N-terminal domain of T [107].

It is relatively common to find in the genome of phages infecting Gram-positive bacteria and mycobacteria holin genes with alternative start codons or two separate holin-like genes [79]. For some it could be demonstrated a pattern of lysis timing regulation compatible with presence of holin and antiholin functions [108,109,110]. For other phages, however, efficient cell lysis seems to require the simultaneous action of the pair of holin-like proteins, and it has been proposed for these cases that the actual holin functional unit might correspond to a complex of two holin effectors [111,112,113].

Holins constitute a very diverse functional group and they were firstly described as small hydrophobic proteins (<150 amino acids), exhibiting at least 1 TMD and a hydrophilic and highly charged C-terminus [84,96]. However, a recent database survey and in silico analysis suggested the presence of 1 TMD as the only minimum requirement for holin function [114]. This study identified 52 families of holin or holin-like proteins, of phage and bacterial origin, the representative members of which have been included in the Transporter Classification Database (TCDB). Based on phylogenetic relationships, 21 of the 52 families could be further grouped into seven superfamilies, each with distinctive characteristics in terms of protein size, number of putative TMDs (up to four), probable membrane topology and organism source distribution [114,115].

### 4.2. Non-Canonical Lysis Systems: Holin-Independent Export of Endolysins

The timing for killing and bursting infected host cells is of major relevance for phage fitness. Delayed lysis may compromise new opportunities of infection, whereas premature burst will negatively impact phage yield [116]. As occurring with most genes, regulation of the expression of lysis functions in time and space can be imposed at the different levels (reviewed in [117]), but the post-translation mechanisms determining the timing of endolysin activity in the CW are of particular importance. Confinement of endolysins in the host cell cytoplasm until holin hole formation (canonical lysis) was for long considered the universal solution to avoid premature end of the virus reproductive cycle. Somewhat surprisingly, however, in 2000 São-José et al. [118] showed that the endolysin of the *Oenococcus oeni* phage fOg44 was synthesized with a typical signal peptide (SP), which directed secretion of the lytic enzyme (Lys44) to the CW compartment by the general secretion system conserved in bacteria (the Sec system). The study also showed that after its synthesis Lys44 was immediately exported to the CW, resulting in the accumulation of the SP-less, mature form of the enzyme in the CW throughout phage development. This obviously raised the question of how premature lysis was prevented and what was the role of the holin in this system. It became later clear that Lys44 was kept inactive in the CW as long as host cells maintained their membrane PMF intact, being quickly activated by drugs that mimicked the PMF-dissipating action of holins [119]. Therefore, although not participating in endolysin release to the CW, the fOg44 holin [120] still retained the key role of defining the lysis timing thanks to its scheduled killing activity. It is yet unknown how the membrane PMF is involved in Lys44 inhibition in the CW. It has been proposed that the lytic enzyme could be controlled by the same mechanisms that regulate host bacteria muralytic enzymes [79,118,119], some of which (autolysins) are well-known for being activated upon PMF collapse [17,121,122].

The Lys44 study and subsequent in silico analysis led to the general perception that several endolysins of both Gram-positive and Gram-negative systems could have export signals, some of which were later confirmed experimentally [3,118,123,124]. Two of such endolysins, Lyz of the coliphage P1 and R^21^ of the lambdoid phage 21 were studied in great detail as prototypes of a new class of lytic enzymes. In contrast to the Lys44 SP, the N-terminal secretion signal of Lyz and R^21^ is not removed by the signal peptidase after Sec-mediated transport to the periplasm. Due to its particular features, the N-terminal TMD remains embedded in the CM after protein translocation, keeping the endolysins anchored to the energized membrane in an inactive state during phage virion production. Endolysins gain their active conformation after being released from the CM, an event that is highly potentiated by the PMF-dissipation action of the cognate holins [125,126,127]. This new signal peptide involved in endolysin export and regulation was initially called signal-arrest-release sequence [125], acquiring later the preferred designation of signal-anchor-release (SAR) domain [127]. After leaving the bilayer, the SAR motif is directly involved in the protein refolding mechanisms that lead to endolysin activation in the periplasm (for details see References [126,127,128]).

Hole formation by cognate holins of SAR endolysins also involves important TMD topological changes in response to PMF dissipation [129]. Yet, in theory, for endolysin activation these holes need only to be large enough to allow passage of ions for PMF collapse. In fact, it was demonstrated that the holes formed by the phage 21 holin (S^21^) were too small to allow passage of the R endolysin of phage λ [130]. Detailed structural and biochemical studies showed that instead of the micron-scale holes formed by the S holin of λ, S^21^ formed channels with a lumen of up to 2 nm [131,132]. For this reason, the term “pinholin” was proposed [130] to differentiate small-hole (pinhole)-forming holins, like S^21^, from canonical holins that form large holes (like the S holin of phage λ). Note, however, that phages engaging the host cell machinery for endolysin export may still encode canonical holins, as verified for phages fOg44 and P1 [80,120].

The production of endolysins with secretion signals (typical SP or SAR) and of pinholins has been meanwhile demonstrated for other phages of Gram-negative and Gram-positive systems [133,134,135]. The mycobacteriophage Ms6 also engages the host Sec system for transport of its endolysin, LysA, to the murein layer. In this system, however, the export signal is conferred by the chaperone-like protein Gp1, which interacts with LysA to promote its Sec-dependent secretion [136,137]. Gp1 homologues are found in several mycobacteriophages [79] and in the case of Ms6 its presence was shown critical for efficient host cell lysis (~70% reduction in the number of released phage particles in absence of Gp1 [136]). Another particularity of the Ms6 lysis system is that the LysA gene encodes two endolysin isoforms as result of an in-frame, alternative translation start site, with the two enzyme variants being required for properly timed and complete cell lysis [138]. Interestingly, in-frame alternative start sites were shown to be responsible for the production of the different subunits that compose some multimeric endolysins [92,93].

More recently, an alternative mechanism of endolysin transport to the murein layer was proposed by Frias et al. [139] that depends neither on holin holes nor on the Sec pathway. The endolysin Svl of the *Streptococcus pneumoniae* phage SV1 has a typical modular organization, exhibiting an N-terminal amidase CD linked to a C-terminal CWBD made of choline binding repeats. The teichoic acids (TAs) of the *S. pneumoniae* CW are decorated with choline residues that serve as ligands for the so-called choline binding proteins, which include PG-degrading enzymes [140]. Genetic analysis and infection of choline-depleted cells led to the conclusion that export and anchoring of Svl to the CW depended only on the presence of choline residues in TAs. Therefore, the authors proposed that Svl, and probably the highly related pneumococcal autolysin LytA, could bind intracellular TA precursors loaded with choline and be co-transported across the CM during their incorporation in the CW.

As shown for the exported endolysins discussed above, the LysA and Svl endolysins accumulate in the CW and their lytic action is somehow restrained by PMF-dependent mechanisms. Again, it was shown that activation of these endolysins requires the membrane-depolarizing action of the cognate holins [136,139]. In the Lys44 study [118] it was speculated that the lethal character of the holin function could also activate and recruit host cell autolysins to assist lysis. This was demonstrated for the pneumococcal autolysin LytA, which upon holin activation contributes significantly to progeny release of phage SV1 [141].

From the description of the different lysis systems just presented it can be easily concluded that, irrespective of the pathway used to deliver endolysins to the CW, one rule seems to have been conserved throughout evolution: endolysins are not allowed to act (or act very poorly) until infected cells are first killed by the holin function. In non-canonical lysis this happens because the endolysins positioned in the CW compartment need to be activated by the membrane-depolarizing action of the holins. In canonical lysis the holin holes are required for endolysin escape to the CW and the physical barrier imposed by the CM has been regarded as the only mechanism that can restrain endolysins from cleaving the PG in this lysis model. In other words, canonical endolysins were considered to accumulate in the cytosol in its fully active conformation, being ready to act as long as contact to the PG is allowed. However, as presented next, recent studies indicate that the PMF-dissipating action of the holin can also activate or potentiate the lytic action of endolysins in canonical lysis.

### 4.3. Endolysin Activation in Canonical Lysis: Beyond Hole Formation

With exception of the secretion signals, exported endolysins (e-endolysins), canonical endolysins (c-endolysins) and autolysins can be highly related regarding their primary sequence and functional domains [85,118,123,142], with the relationship between membrane PMF and lytic activity being well established for e-endolysins and several autolysins [17,79,80,122,143]. Considering this, and the fact that in all known lysis systems holin-mediated cell killing precedes always endolysin action, Proença et al. [144] hypothesized that PMF collapse by holins could also sensitize bacteria to the lytic action of c-endolysins. This effect would be naturally masked by the essential role of the holin holes in c-endolysin release to the CW. This type of endolysin “activation” in canonical lysis would fit several observations indicating that when bacteria are under conditions supporting robust growth (and PMF), they are much less susceptible to the lytic action of recombinantly produced c-endolysins added from without [144,145]. The fact that the lytic activity of some c-endolysins was drastically enhanced by CM drugs mimicking the PMF-dissipating role of holins agreed also with the idea that fully energized cells are capable of counteracting the lytic enzymes, at least to some extent [144,146].

A role of holins in endolysin activation in canonical lysis gained further support after recent studies with the c-endolysins LysSPP1 and Lys11, which are produced by the *Bacillus subtilis* and *S. aureus* phages SPP1 and phi11, respectively [147]. The investigation showed that conditions inducing CM depolarization (holin action, membrane ionophores or nutrient depletion) dramatically increased the susceptibility of host bacteria to the lytic action of the c-endolysins. In one key experiment the native LysSPP1 was transformed into an e-endolysin by fusing to the enzyme’s N-terminus a host cell Sec-type SP. The fusion was inserted in a plasmid that allowed controlled expression of SP-LysSPP1 during *B. subtilis* growth. As expected, the SP element directed the secretion of the artificial e-endolysin to the CW, with consequent accumulation of the processed form corresponding to the native c-endolysin. Despite the high levels of LysSPP1 accumulation in the CW, *B. subtilis* cells continued to grow basically unaffected, indicating that c-endolysin activity was being restrained after translocation and SP removal. Moreover, addition to these cells of a PMF-dissipating agent immediately elicited endolysin activity and cell lysis, in striking similarity to the general lysis features of known e-endolysins.

**Figure 4:**
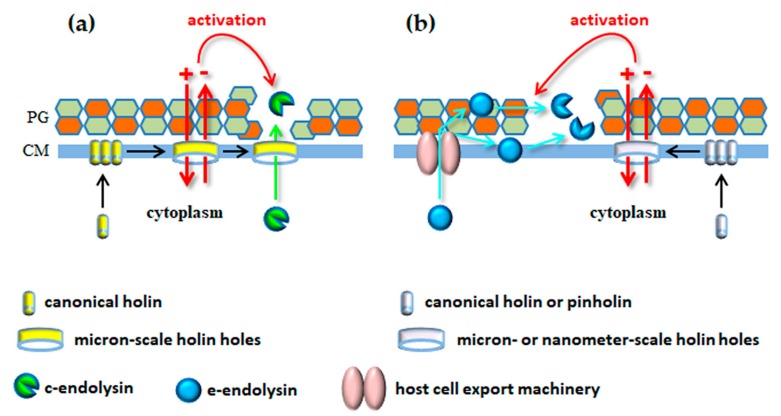
Key features of canonical (**a**) and non-canonical (**b**) lysis models. In the canonical model endolysins (c-endolysins) can only gain access to the CW compartment through the holin holes, which need therefore to be large enough to accommodate the enzymes’ size. In non-canonical lysis, host cell machineries (e.g., the bacterial Sec system) export the lytic enzymes (e-endolysins) to the extracytoplasmic space, where they are maintained inactive in association with the CW or the CM. Dissipation of the membrane PMF by the holin holes (canonical holins or pinholins) is an essential requirement for activation of e-endolysins. Recent evidences indicate that holin-mediated PMF collapse may also potentiate the lytic activity of c-endolysins (see text). CM, cytoplasmic membrane; PG, peptidoglycan.

In conclusion, it seems that holins have two interconnected functions in bacterial lysis mediated by dsDNA phages, independently of the pathway used to deliver endolysins to the CW. One is to determine the end of phage infection by inducing cell death and the other is to activate/boost endolysin lytic activity as result of PMF dissipation. A third essential role, which is that of providing a conduit for endolysin access to the CW, is exclusive of canonical lysis systems (Figure 4). As mentioned in Section 4.1, the presence of a CWBD in endolysins may keep the lytic enzymes associated to CW debris after host cell lysis, avoiding by this way collateral effects on potential new hosts in the vicinity, particularly if these lack an OM protecting the PG layer. The capacity of growing cells to counteract c-endolysins attacking from the outside may provide an additional strategy to spare potential hosts, assuming that some endolysins can still diffuse to the medium after lysis of phage-infected cells. It should be noted nonetheless that the study of Fernandes and São-José [147] also highlighted that the capacity of energized cells to restrain c-endolysin activity may vary significantly depending on growth conditions and on the tested bacterium/endolysin pair.

### 4.4. Overcoming the Last Barrier

In the previous sections we saw that dsDNA phages use the holin and endolysin functions as core elements of different strategies to lyse bacterial hosts. These phages can nevertheless encode other lysis products, some of which were recently revealed crucial for efficient host cell burst. The best example that comes from early studies is provided by the Rz and Rz1 proteins of phage λ. The *Rz* and *Rz1* genes locate immediately downstream of the *S* and *R* dyad, with *Rz1* being fully embedded in *Rz* in a +1 frame, and with the four genes actually forming the complete λ lysis cassette [80]. Rz is a CM protein with a periplasmic domain, whereas Rz1 is an OM lipoprotein [148,149,150]. Rz and Rz1 were initially described as auxiliary functions because they appeared to be required for efficient lysis only when the OM was stabilized by millimolar concentrations of divalent cations, namely Mg^2+^ [151].

In 2007, an in-depth phage genome database survey performed by Summer et al. [152] showed that a large fraction of phages infecting Gram-negative bacteria encoded Rz and Rz1 equivalents, with most of the corresponding genes being arranged in three possible ways (embedded, overlapped or separate). The level of conservation of these proteins in phage genomes strongly indicated that they fulfilled a role in lysis far more important than just being auxiliary functions. In addition, the study revealed a major clue for the function of the Rz/Rz1 pair because the *E. coli* phage T1 (and few other phages) appeared to encode a single protein (gp11) sharing the features of both proteins, meaning that gp11 would be simultaneously anchored to the CM and OM. This strongly suggested that Rz and Rz1 interacted, as previously indicated for the phage T7 equivalents [153], to bridge the CM and OM while spanning the entire periplasm. Such observation inspired the term “spanins” to designate this functional class of proteins, with the inner membrane (CM) component (Rz) named i-spanin and the OM component (Rz1) called o-spanin [152].

The phage λ Rz and Rz1 subcellular localization and the mode of interaction of their C-terminal portions in the periplasm have been confirmed and studied in detail (for a review see [80]). Moreover, video-microscopic studies of λ lysogens lysis phenotypes showed that, depending on the physiological conditions, the absence of the spanin function could result in lysis blockage after the holin trigger and PG degradation. For example, in absence of the artificial shearing forces resulting from culture flask agitation under aerobic growth, cells acquired a spherical shape and became apparently stabilized by the OM, resisting osmotic lysis, independently of the presence of divalent cations in the medium [154]. The mechanism by which spanins achieve elimination the OM barrier most likely involves CM-OM fusion. In the current model for the two-component spanins, the Rz/Rz1 heterotetrameric complexes connecting the CM and OM accumulate in the periplasm during phage infection, being accommodated within the gaps formed by the PG cross-linking. Destruction of the PG by the endolysin releases the spanins for oligomerization by lateral diffusion, providing the free energy for the Rz coiled-coil structures to change to a collapsed conformation. This forces the opposing membranes to come into contact, stimulating their fusion and destabilization (for mechanistic details see [80,155,156]).

In summary, the available data indicates that under infection conditions that are perhaps closer to those found in nature, the spanin function is essential for lysis in phage λ, and probably for many phages infecting Gram-negative bacteria. In this scenario, phage-mediated lysis of Gram-negative hosts can be viewed as a pathway of three sequential steps that once initiated becomes irreversible [80]. The first step corresponds to CM permeabilization by the holin that leads to PG degradation by the endolysin (second step), which in turn liberates the spanin to promote OM disruption (final step). Despite forming a sequential pathway, holins, endolysins and spanins are mechanistically independent, that is, they do not need to physically interact to accomplish their role in lysis.

As described in Section 2, the cell envelope of mycobacteria has a mycolic acid-rich OM covalently attached to the arabinogalactan–peptidoglycan network. It could be expected, therefore, that phages infecting mycobacteria would encode functional analogues of spanins. Actually, a third lysis function called LysB is encoded by almost all known mycobacteriophages, which is specifically designed to attack the particular mycobacterial OM [79,157,158]. LysB enzymes were shown to act in different lipid substrates, but their esterase activity towards the ester linkages that connect mycolic acids to arabinogalactan was considered the preponderant action responsible for elimination of the OM barrier [157,158,159]. Mutant Ms6 and Giles mycobacteriophages depleted of the LysB function were shown viable under laboratory conditions. However, they were defective regarding completion of host cell lysis (timing and lysis progression also affected in Giles), which translated in a considerable fraction of the virion progeny being trapped within deficiently lysed host cells [158,160]. LysB homologues were identified in phages infecting *Rhodococcus equi* and *Gordonia* spp., which present also mycolic acids in their BCE [161,162]. It is presently unknown how LysB proteins reach their substrates, but it is possible that they simply diffuse to the outermost layers of the BCE after holin/endolysin disruption of the CM and PG barriers [79].

## 5. Conclusions

Virus-host cell interactions are characterized by a continuous arms race for survival. Bacteria try to protect themselves from environmental insults, like toxic chemicals and infection agents, by producing a rather impermeable cell envelope. In its most complex configuration, the BCE can be composed of two membranes separated by a CW, plus an extracellular protective coat made of polymeric substances, like for example a polysaccharidic capsule. Tailed phages are highly sophisticated nanomachines that have evolved tools and mechanisms to efficiently overcome all the BCE barriers at two crucial steps of their replicative cycle: virus entry to bacteria and virion offspring escape from infected cells. For the entry step, phages use tail receptor-binding proteins to attach to components (receptors) of the BCE, some of which may be degraded during the binding process. A remarkable example of this is provided by certain podoviruses, which employ tailspikes with depolymerase activity to bind and cleave extracellular (capsule) or structural (LPS) polysaccharides. Upon irreversible binding, most phages use their tail dynamics to penetrate the different layers of the BCE and to deliver the viral DNA into the bacterial cytoplasm. Insertion of the tail substructures across the CW can be assisted by virion-associated lysins that promote a local degradation of the PG network.

While phage tail penetration and DNA delivery across the BCE needs to occur without compromising host cell viability, successful virion progeny release to the extracellular medium usually requires abrupt and massive destruction of all cell envelope barriers. Our current knowledge of lysis systems suggests that tailed phages acquired functions and evolved mechanisms in response to the complexity of the host BCE. The minimal lysis system seems to require a holin and an endolysin to disrupt the CM and the CW, respectively. This dual system is usually sufficient to achieve osmotic lysis of host cells with only these two barriers (Gram-positive bacteria). However, host bacteria with an OM (Gram-negative and mycobacteria) may display incomplete lysis, or even fail to burst, in presence of such minimal lytic system. Therefore, in addition to the holin/endolysin pair, the majority of phages infecting these hosts employ additional functions to overcome the last barrier of the BCE (spanins in Gram-negative bacteria and lipolytic enzymes in mycobacteria). The latest developments in the field have also reinforced the crucial role of the holin function in lysis. It has been found that, irrespective of the pathway followed to deliver the endolysins to the CW compartment (holin-dependent or -independent), the holin-mediated PMF dissipation seems to fulfil the key role of activating or of boosting the lytic activity of endolysins in the CW.
